# The Perme scale score as a predictor of functional status and complications after discharge from the intensive care unit in patients undergoing liver transplantation

**DOI:** 10.5935/0103-507X.20190016

**Published:** 2019

**Authors:** Camila Santos Pereira, Alexandra Torres de Carvalho, Adriane Dal Bosco, Luiz Alberto Forgiarini Júnior

**Affiliations:** 1 Centro Universitário Metodista IPA - Porto Alegre (RS), Brasil.; 2 Complexo Hospitalar Irmandade Santa Casa de Misericórdia de Porto Alegre - Porto Alegre (RS), Brasil.; 3 Curso de Fisioterapia e Programa de Pós-Graduação em Saúde e Desenvolvimento Humano, Universidade La Salle - Canoas (RS), Brasil.

**Keywords:** Liver transplantation, Postoperative complications, Muscle strength, Pain measurement, Dyspnea, Mobility limitation

## Abstract

**Objective:**

To assess the Perme mobility scale score as a predictor of functional status and complications in the postoperative period in patients undergoing liver transplantation.

**Methods:**

The sample consisted of 30 patients who underwent liver transplantation. The patients were evaluated at two time points to determine their perception of pain, degree of dyspnea, peripheral muscle strength, and functional status according to the Perme scale. The collected data were analyzed by descriptive and inferential statistics. To compare the means between the evaluations, Student's *t* test for paired samples was applied. In case of asymmetry, the Wilcoxon test was used. In the evaluation of the association between the quantitative variables, the Pearson or Spearman correlation tests were applied.

**Results:**

A total of 30 individuals who underwent liver transplantation were included. The patients were predominantly male, and the mean age was 58.4 ± 9.9 years. The most prevalent underlying pathology was cirrhosis C virus (23.3%). Significant associations of the time on mechanical ventilation with the Perme scale score at discharge from the intensive care unit (r = -0.374; p = 0.042) and the number of physical therapy treatments (r = -0.578; p = 0.001) were recorded. When comparing the results of the initial evaluation and the evaluation at hospital discharge, there was a significant improvement in functional status (p < 0.001).

**Conclusion:**

Functional mobility, peripheral muscle strength, pain perception, and dyspnea are significantly improved at hospital discharge compared with those at inpatient unit admission.

## INTRODUCTION

Liver transplantation (LTx) is the most commonly indicated treatment for patients with end-stage liver disease, as it allows longer survival for these individuals.^(^^1^^)^ Due to advances in medicine, the development of new surgical techniques, advances in the use of immunosuppression, and types of anesthesia available, organ and tissue transplantation is currently a safe and efficient therapeutic option for the treatment of terminal diseases, promoting improvement in quality of life and life expectancy.^(^^2^^)^

The indications for LTx are well established globally, and patients undergoing transplantation should be properly selected. LTx should only be indicated when conventional therapeutic methods have been exhausted and the likelihood of survival and quality of life are higher with LTx.^(^^3^^)^ Organ transplantation is indicated in some cases of liver disease complications. In adults, the most common indications for LTx are chronic hepatitis B or C, alcoholic liver disease, primary biliary cirrhosis, sclerosing cholangitis, and autoimmune hepatitis.^(^^4^^)^

Some studies have investigated the functional status, exercise capacity, and respiratory muscle strength in patients with chronic liver disease, and these patients have been shown to have reduced muscle mass, decreased exercise capacity, and muscle weakness, resulting in functional losses that may interfere with the course of the disease, leading to postoperative complications and reducing survival after transplantation.^(^^5-8^^)^ After LTx, patients are admitted to the intensive care unit (ICU). This postoperative period is characterized as a critical phase, which demands special care due to the particularities of the multisystemic changes resulting from liver disease and the lack of liver function in the postoperative period.^(^^9^^)^ Several studies have shown the occurrence of disorders resulting from prolonged bed rest during ICU stay, and the consequences may persist for many years after discharge.^(^^10,11^^)^

Considering the need for careful patient evaluation to establish advanced treatment strategies in this period, Perme et al. developed an instrument called the Perme Intensive Care Unit Mobility Score to measure patient mobility in a quick, objective, and specific manner.^(^^12^^)^

The objective of this study was to evaluate the Perme scale as a predictor of functional status and complications after discharge from the ICU in patients undergoing LTx and to evaluate and analyze its correlation with the length of ICU stay, time on mechanical ventilation (MV), length of hospital stay, pain, dyspnea, and peripheral muscle strength.

## METHODS

This study was a prospective, observational study conducted at *Hospital Dom Vicente Scherer*, belonging to *Complexo Hospitalar Santa Casa de Porto Alegre* (CHSCPA), from September 2017 to March 2018. Patients of both sexes aged 18 years or older who underwent LTx were included.

Patients who presented with encephalopathy, mental confusion, hemodynamic instability, or generalized swelling and who had complications due to evisceration in the late postoperative period or who were not prescribed physical therapy were excluded from the study. Thus, 33 patients were initially included, 3 of whom were excluded (2 due to death and 1 due to hemodynamic instability). This study, in compliance with resolution 466/12, was submitted to and approved by the Ethics and Research Committee of *Irmandade Santa Casa de Misericórdia de Porto Alegre* under number 2,262,095. All the participants were invited to participate, and those who agreed to participate signed an informed consent form.

For data collection, patients were evaluated according to the care routines of the physical therapy service in the postoperative period of LTx at two time points to determine pain perception, degree of dyspnea, peripheral muscle strength, and functional status/mobility. Prior to the evaluation, the data related to the characteristics of the sample were recorded on a specific evaluation form, always by the same evaluator. This assessment was applied on the first day in the inpatient unit (IU). After the evaluation, the care routine of the physical therapy service was started, and the patient was evaluated again at prehospital discharge.

The Visual Analogue Scale (VAS), an easy-to-apply one-dimensional instrument validated in Brazil that was applied to evaluate pain perception levels, with the patient indicating the degree of pain felt at the time, was considered an adequate method for estimating pain intensity.^(^^13^^)^ The modified Borg scale was used to evaluate the degree of dyspnea and required the participants to answer questions about the sensation of breathlessness (dyspnea), quantified on a scale ranging from zero (no dyspnea) to 10 (maximal dyspnea).^(^^14^^)^

Peripheral muscle strength was measured using the Medical Research Council (MRC) sum-score. With patients sitting with their feet comfortably supported, the bilateral muscle strength of the following muscle groups was assessed and scored: shoulder abductors, elbow flexors, wrist extensors, hip flexors, knee extensors, and dorsiflexors. Muscle strength was scored from 0 to 5, with 0 corresponding to the absence of muscle contraction and 5 corresponding to normal muscle strength.^(^^15^^)^ Each patient's mobility status was measured by the Perme scale, which aims to determine the functional mobility status of the patient by evaluating 15 items grouped into 7 categories: mental status, potential mobility barriers, functional strength, bed mobility, transfers, gait, and endurance. The score ranges from 0 to 32 points; the higher the score is, the lower the need for assistance.^(^^12^^)^

The data collected were analyzed by descriptive and inferential statistics using means and standard deviations, and asymmetric data were analyzed using medians and interquartile ranges. Categorical variables are expressed in absolute and relative frequencies. To compare means between the evaluations, Student's *t* test for paired samples was applied. The Wilcoxon test was used for asymmetric data. In the evaluation of the association between the quantitative variables, the Pearson or Spearman correlation test was applied, using the Statistical Package for the Social Sciences (SPSS), version 21.0. In all cases, differences were considered significant when p < 0.05.

## RESULTS

During the period from September 2017 to March 2018, 30 individuals who underwent LTx were included. There was a predominance of male patients, and the mean age was 58.4 ± 9.9 years. The most prevalent underlying pathology was cirrhosis C virus (HCV; 23.3%). The median time on MV was 675 minutes, and the length of hospital stay was 18 days. The other sample characteristics are described in table 1.

**Table 1:** Characteristics of the patient sample

Variables	Results n = 30
Age (years)	58.4 ± 9.9
Sex	
Male	26 (86.7)
Underlying disease	
HCV	7 (23.3)
HBV	2 (6.7)
HCV + HCC	5 (16.7)
Cirrhosis by NASH	4 (13.3)
Other	12 (39.9)
MELD	15.6 ± 6.1
Length of ICU stay (days)	5 (4 - 6)
Length of hospital stay (days)	18 (15 - 25)
Time on MV (minutes)	675 (557 - 1106)
Perme score at ICU admission	5.5 (1 - 9)
Perme score at ICU discharge	23.6 ± 5.3
Perme score in the IU	28.2 ± 5
Perme score at hospital discharge	31.7 ± 0.7
Number of physical therapy treatments	34.9 ± 17.4

HCV - hepatitis C; HBV - hepatitis B; HCC - hepatocellular carcinoma; MELD - Model for End Stage Liver Disease; ICU - intensive care unit; MV - mechanical ventilation; IU - inpatient unit. The results are expressed as the means ± standard deviation, n (%) or median.

When comparing the results of the initial evaluation with those at hospital discharge regarding dyspnea and pain, a significant reduction in these variables was observed (Table 2^)^). There was also an increase in peripheral muscle strength and in functional mobility, with better results obtained at hospital discharge. Regarding the MRC score, we observed that three patients presented a score lower than 48, indicating ICU-acquired muscle weakness (ICUAW).

**Table 2:** Comparison between the initial and final evaluations

Variables	IU admission	Hospital discharge	p value
VAS	2 (1 - 5)	0 (0 - 1)	0.001
BORG	0.5 (0 - 1)	0 (0 - 0)	0.002
Perme	28.2 ± 5.0	31.7 ± 0.7	< 0.001

IU - inpatient unit; VAS - Visual Analogue Scale; BORG - Modified Borg scale; Perme - Perme mobility scale; MRC - Medical Research Council. The results are expressed as the median or mean ± standard deviation.

Table 2^)^ presents a comparison of the Perme scale score at the different time points (ICU admission and discharge), indicating significantly better results at hospital discharge (p < 0.001) compared with the initial evaluation.

With regard to the association of the Perme scale scores with the clinical variables, table 3 shows a significant correlation (p < 0.05) between the Perme scale score at ICU discharge and time on MV. Specifically, the longer the duration of MV was, the lower the Perme score at ICU discharge. When the association of the Perme score at ICU discharge with the length of hospital stay and the length of ICU stay was assessed, there was no significant correlation.

**Table 3:** Association between the Perme mobility scale score and demographic and clinical variables

Variables	Perme at ICU discharge		Perme in the IU	
	r	p value	r	p value
Length of ICU stay (days)		0.535		0.420
Length of hospital stay (days)		0.551		0,141
Time on MV (minutes)	-0.374	0.042*		0.450

ICU - intensive care unit; IU - inpatient unit; MV - mechanical ventilation.

* Significant association between the time on mechanical ventilation and the Perme score at discharge from the intensive care unit.

In this study, a significant inverse association was observed between the number of physical therapy treatments and the Perme score in the IU (r = -0.578; p = 0.001); specifically, the lower the Perme score was, the greater the number of physical therapy treatments performed.

## DISCUSSION

This study demonstrated that most of the individuals evaluated were discharged from the hospital with improved functional mobility and peripheral muscle strength, as well as a decreased sensation of dyspnea and pain. There was no association between the functional status at ICU discharge and the length of ICU or hospital stay; however, there was an association with time on MV.

Due to the general condition of patients with chronic liver disease in the preoperative period and the complexity of the surgical intervention, a large proportion of global complications occur in the postoperative period of LTx, which include important changes in functional capacity.^(^^5^^)^

In our findings regarding the sample characteristics, the mean age was 58.4 ± 9.9 years, i.e., similar to the mean age identified in the current literature.^(^^16^^)^ The patients were predominantly male, with men comprising 86.7% of the sample, corroborating the characteristics of the sample reported by Schraiber et al. In their study with 206 patients with cirrhosis and hepatocellular carcinoma undergoing LTx, the mean patient age was 56.3 ± 7.3 years.^(^^17^^)^ Among the pathologies that caused the LTx, the most frequent was cirrhosis due to HCV, present in 23.3% of the patients, which is currently one of the main causes of chronic liver disease in the world.^(^^18^^)^

Since 2006, the donor liver allocation system for LTx in Brazil has been administered according to the criterion of disease severity, based on the Model for End-Stage Liver Disease (MELD) score, regardless of the amount of time on the waiting list.^(^^4^^)^ Regarding the liver disease severity score, the study participants presented a mean MELD score of 15.6 ± 6.1, corroborating our findings. Galant et al.,^(^^5^^)^ in a study consisting of 86 patients awaiting LTx, found a mean MELD score of 16 ± 2 in the group diagnosed with HCV. Another study by Galant et al.^(^^19^^)^ found an inverse correlation between the MELD score and the distance walked in the 6-minute walk test (6MWT), suggesting that the score can be used as a predictive variable of functional status in patients who are candidates for LTx.

Regarding the length of hospital stay, we observed a median time of 18 days, which is similar to that found in a retrospective study performed in cirrhotic patients undergoing LTx (19 days).^(^^20^^)^

In a prospective cohort study, the authors evaluated and compared, among other variables, the length of ICU stay and the length of hospital stay after LTx in patients with and without a diagnosis of hepatopulmonary syndrome. They identified that patients diagnosed with hepatopulmonary syndrome remained in the hospital longer (24.1 ± 4.3 days *versus* 20.2 ± 3.9 days).^(^^21^^)^ Thus, the length of hospital stay may vary according to the demographic characteristics of patients and their comorbidities.

According to França et al.,^(^^22^^)^ some factors may contribute to the impairment of peripheral muscle strength, especially bed rest, sepsis, and MV, which, in turn, may lead to a longer duration of orotracheal intubation and longer hospital stay. Patients hospitalized in the ICU for more than 7 days may present an important degree of peripheral and/or respiratory muscle weakness.^(^^23,24^^)^

Peripheral muscle strength was assessed according to the MRC criteria, with a total score below 48 indicating ICUAW.^(^^25^^)^ In our study, three of the evaluated participants had a score of less than 48 on admission to the IU, suggesting an ICUAW diagnosis, which is a prognostic factor for longer hospital stay and risk of mortality after hospital discharge.^(^^26^^)^

When the patients were evaluated at hospital discharge, an increase in the MRC score was found, demonstrating improvement in peripheral muscle strength. The study participants' assistance by a physical therapy team during the period of hospital stay may have contributed to this improvement. As suggested by existing clinical trials, actions aimed at encouraging activity and early mobilization in the ICU can reduce the duration of MV, length of ICU stay, and length of hospital stay and improve physical function while maintaining muscle strength.^(^^27-29^^)^

In the functional evaluation using the Perme scale, we found a significant score improvement from ICU admission to hospital discharge. Several scales have been used, both in clinical practice and in research with patients hospitalized in the ICU or IU.^(^^26,30,31^^)^

Borges et al.^(^^32^^)^ evaluated the functional level in patients undergoing cardiac surgery using the functional independence measure (FIM) scale and found that the functional level was lower on the seventh postoperative day compared with the level at hospital discharge, with significant changes in the performance of activities of daily living. This result is similar to the findings of Santos et al.,^(^^33^^)^ who analyzed the functional progression of critically ill patients admitted to the ICU, showing significant progress in functional performance at hospital discharge. A recent study evaluated the functional status of patients within 48 hours after ICU discharge, using the Barthel and Katz indices, and concluded that both were able to detect functional impairment after discharge from the ICU. Furthermore, the results suggested superiority of the first index because it was better able to assess the ability to perform the tasks.^(^^34^^)^

We demonstrated a significant association between time on MV and the Perme scale score at ICU discharge; namely, the longer the time on MV was, the lower the Perme score at ICU discharge. The explanation for this finding is that one of the most important factors related to the impairment of functional independence is the length of ICU stay, as well as the duration of MV.^(^^26^^)^

When we correlated the Perme score in the IU with the number of physical therapy treatments, we found a significant inverse association. The patients who presented worse functional mobility after discharge from the ICU were those who received the most care from the physical therapy service. It is important to note that all the patients enrolled in the study received physical therapy care, during both the time they were in the ICU and the time they were in the IU, as physical therapy is one of the factors that can alter the functional outcome of patients.^(^^26-29^^)^

Among the limitations of this study, it is necessary to highlight the need for more studies on the subject, as difficulties were found in analyzing the results, limiting the possibility of comparison with the literature. It seems that more studies and clinical trials with liver transplant patients are needed, as a well-designed intervention may affect the postoperative period, avoiding complications and even reducing costs associated with prolonged hospital stays.

## CONCLUSION

There was improvement in dyspnea, peripheral muscle strength, functional mobility, and pain perception when comparing the evaluations performed in the inpatient unit and at hospital discharge. Time on mechanical ventilation was associated with functional status at hospital discharge, and the Perme scale score was associated with the length of stay in the inpatient unit.
